# Direct observation and imaging of a spin-wave soliton with *p*-like symmetry

**DOI:** 10.1038/ncomms9889

**Published:** 2015-11-16

**Authors:** S. Bonetti, R. Kukreja, Z. Chen, F. Macià, J. M. Hernàndez, A. Eklund, D. Backes, J. Frisch, J. Katine, G. Malm, S. Urazhdin, A. D. Kent, J. Stöhr, H. Ohldag, H. A. Dürr

**Affiliations:** 1 Department of Physics, Stanford University, Stanford, California 94305, USA; 2 Stanford Institute for Materials and Energy Sciences, SLAC National Accelerator Laboratory, 2575 Sand Hill Road, Menlo Park, California 94025, USA; 3 Department of Materials Science and Engineering, Stanford University, Stanford, California 94305, USA; 4 Grup de Magnetisme, Departament de Física Fonamental, Universitat de Barcelona, Barcelon 08028, Spain; 5 Integrated Devices and Circuits, School of Information and Communication Technology, KTH Royal Institute of Technology, Kista 16440, Sweden; 6 Department of Physics, New York University, 4 Washington Place, New York, New York 10003, USA; 7 Advanced Instrumentation for Research Division, SLAC National Accelerator Laboratory, 2575 Sand Hill Road, Menlo Park, California 94025, USA; 8 HGST, a Western Digital Company, 3403 Yerba Buena Road, San Jose, California 95135, USA; 9 Department of Physics, Emory University, 201 Dowman Drive, Atlanta, Georgia 30322, USA; 10 Stanford Synchrotron Radiation Laboratory, SLAC National Accelerator Laboratory, 2575 Sand Hill Road, Menlo Park, California 94025, USA

## Abstract

Spin waves, the collective excitations of spins, can emerge as nonlinear solitons at the nanoscale when excited by an electrical current from a nanocontact. These solitons are expected to have essentially cylindrical symmetry (that is, *s*-like), but no direct experimental observation exists to confirm this picture. Using a high-sensitivity time-resolved magnetic X-ray microscopy with 50 ps temporal resolution and 35 nm spatial resolution, we are able to create a real-space spin-wave movie and observe the emergence of a localized soliton with a nodal line, that is, with *p*-like symmetry. Micromagnetic simulations explain the measurements and reveal that the symmetry of the soliton can be controlled by magnetic fields. Our results broaden the understanding of spin-wave dynamics at the nanoscale, with implications for the design of magnetic nanodevices.

## Abstract (web summary)

Injecting spin-polarized current into a ferromagnetic thin film via a nanocontact is expected to generate a radially-symmetric spin wave soliton. Here, the authors use time-resolved x-ray microscopy and micromagnetic simulations to demonstrate the occurrence of *p*-like symmetry associated with non-uniform magnetic fields in the nanocontact region.

## Graphical abstract

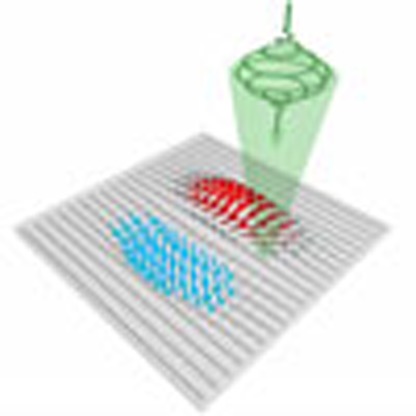

In magnetic materials, the electrons' spin couples to form collective magnetic excitations called spin waves. Such spin waves are the building blocks of novel magnetic nanodevices^1^ to transmit signals at room temperature^2^, or to encode information^3^, offering a potential pathway towards future electronics. Historically, the manipulation of spin waves required spatially extended microwave magnetic fields, limiting the scalability towards small devices. However, the recent discovery of alternative physical mechanisms for spin-wave excitation based on the use of electric currents, most prominently the spin torque transfer^4^,^5^ and the spin Hall effect^6^,^7^,^8^, promises novel ways to achieve nanoscale control of spin waves.

It is now established that the local injection of strong spin-polarized electrical currents can generate nonlinear spin waves with both itinerant^9^,^10^,^11^,^12^,^13^ and localized^11^,^14^,^15^,^16^,^17^,^18^ character. This character is determined by the relative orientation between the material internal field and the applied external field. Spin waves of both characters are also required to preserve the radial symmetry of the nanocontact used to inject the spin-polarized current. Such radial symmetry can be perturbed by the Oersted field generated by the current flowing through the nano-contact, however, with qualitatively different effects for itinerant and localized excitations. In case of itinerant spin waves, excited when a magnetic field saturates the magnetization out of the plane of the sample, the Oersted field does not break the in-plane symmetry of the spin-wave precession. Hence, it is expected that the spin waves form a circular pattern far away from the nano-contact^9^. This type of excitation has been reported with micro-focused Brillouin Light Scattering^12^,^13^. For the case of localized excitations, created when an in-plane magnetic field is applied to the sample, the Oersted field does break the in-plane symmetry, and a spatial shift of the excitation away from the nano-contact has been predicted by numerical simulations^19^,^20^,^21^. However, an experimental visualization of localized excitations has been hampered by the lack of a suitable imaging technique combining spatial and temporal resolution with magnetic sensitivity. Therefore, the spatial properties of localized solitons are currently unknown. For instance, it is unclear whether they can only possess the full radial symmetry (*s*-like) or if excitations with different symmetry (*p*-like) are also allowed^22^, as shown schematically in Fig. 1c.

Here we probe the current-induced nonlinear spin-wave excitations via time-resolved X-ray magnetic circular dichroism (XMCD)^23^ using a scanning transmission X-ray microscope as described in Fig. 1. We directly image the nanoscale motion of localized nonlinear spin waves in the magnetic layer below a nanocontact with 50 ps temporal resolution, hence creating a spin-wave movie. Our results reveal the existence of a localized spin-wave soliton characterized by a nodal line (that is, with *p*-like symmetry). Micromagnetic simulations reproduce this *p*-like soliton and also demonstrate a transition to *s*-like symmetry with increasing confinement.

## Results

### X-ray microscopy

**Figure 1:**
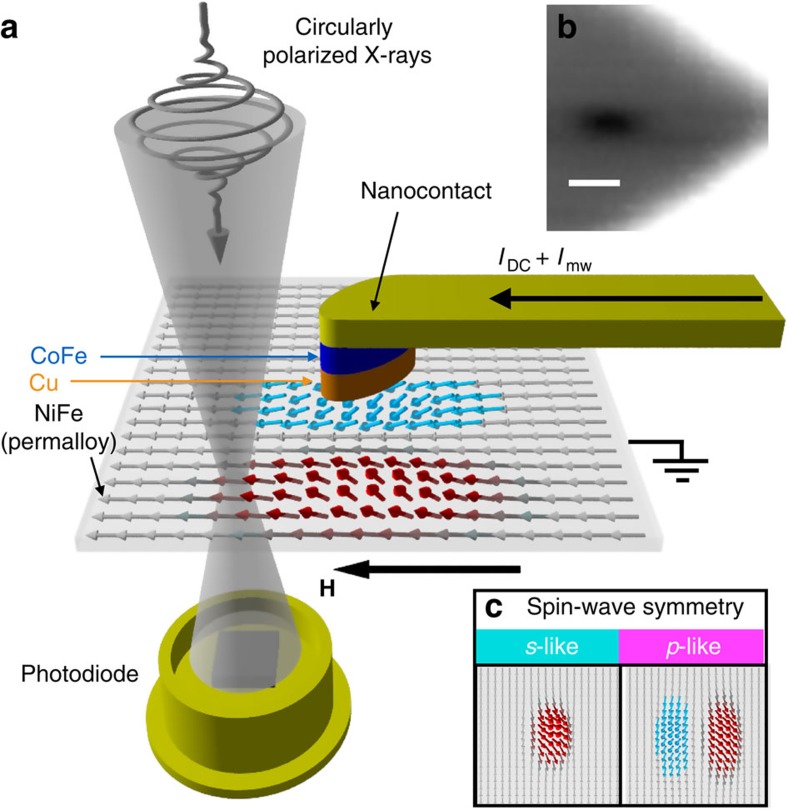
Overview of the experiment. (**a**) Schematic of the measurement and of the sample. The circularly polarized X-rays generated at the elliptically polarizing undulator at beamline 13 at the Stanford Synchrotron Radiation Lightsource (SSRL) are focused to a 35-nm spot using a zone-plate, determining the spatial resolution. The sample comprises a NiFe(5 nm)/Cu(4 nm)/CoFe(8 nm) multilayer, where the Cu and CoFe layer are patterned into an ellipse of 150 × 50 nm^2^, whereas the NiFe layer is a larger mesa. Spin waves are excited when a magnetic field **H** is applied in the sample plane, and a direct current *I*_DC_ flows into the nanocontact. A microwave current *I*_mw_ is superimposed to the direct current to synchronize the spin-wave excitation with the X-ray detection and SSRL's master clock. The time-resolved variation of the magnetization along the X-ray propagation direction is probed by XMCD, measured with an avalanche photodiode as the variation of the signal transmitted through the sample. (**b**) X-ray image showing the topography of the sample. Scale bar, 200 nm. (**c**) Schematic representation of two types of spin wave symmetries.

The schematic of the sample and of the measurement is shown in Fig. 1a. When electrons flow from the permalloy film through the nanocontact to the CoFe layer, spin accumulation allows one spin polarization to pass. Electrons of the other spin polarization are reflected at the Cu/CoFe interface back into the permalloy layer. In the geometry of Fig. 1, these reflected spins transfer their spin angular momentum to the permalloy layer via the spin torque effect^4^,^5^. This torque acts as to increase the relative angle between the CoFe and NiFe magnetizations. This is the mechanism that, as long as the current is on, drives the emission of spin waves. We characterized the spin-wave emission with a spectrum analyzer while the sample was mounted on the X-ray microscope. We observed a frequency red-shift with current characteristic for a localized spin-wave excitation (see Supplementary Fig. 1 for details).

Figure 1b shows an X-ray image of the Cu/CoFe nanocontact (black), the Au electrical connections (grey) and the permalloy film (white). It was obtained with the X-ray energy tuned to the L_3_ absorption edge of Ni (852.7 eV). To probe the magnetic properties of the permalloy layer, we switch the X-ray polarization from linear to circular, keeping the energy fixed at the Ni L_3_ edge. In this condition, the XMCD^23^ probes the component of the Ni magnetization along the X-ray incidence direction (perpendicular to the permalloy layer plane). Without any current this perpendicular magnetization component is zero as the sample is magnetized in the film plane by an applied static magnetic field *μ*_0_**H**=60 mT. However, the excitation of spin waves generate an oscillating magnetization component that is measured by the time-resolved XMCD. XMCD images are collected using a high-sensitivity and high-frequency quasi-stroboscopic technique developed for these measurements (see Methods for details). This technique allows us to synchronize the spin-wave phase with the X-ray pulses via current injection locking. Stroboscopic images are collected for six phases of the magnetization precession each 60° apart, corresponding to a time delay of 27 ps.

**Figure 2:**
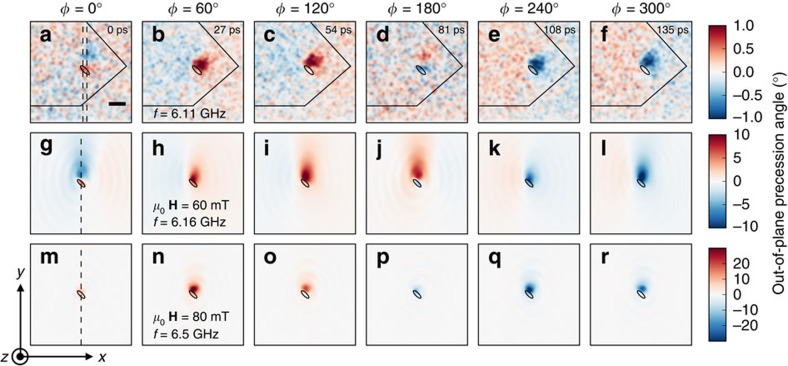
Experimental and simulated results. (**a**–**f**) Experimental time-resolved magnetization precession angle around a nanocontact spin torque oscillator (black open ellipse) measured with a scanning transmission X-ray microscope with a *μ*_0_**H**=60 mT magnetic field applied parallel to the *x* axis. The six images are 1.5 × 1.5 μm^2^ spatial maps, representing snapshots of the magnetization dynamics with a relative time difference of 27 ps. The black solid lines are a schematic representation of the electrical contacts of the sample. Scale bar, 200 nm. Simulated spatial maps of the magnetization precession for applied fields (**g**–**l**) *μ*_0_**H**=60 mT and (**m**–**r**) *μ*_0_**H**=80 mT. The dashed lines indicate the location where vertical cross-sections of the images was calculated, as discussed in the main text. The colour scheme is qualitatively the same for all plots, but it is quantified differently for each rows by the respective colourbar on the right side of the figure.

The resulting XMCD images of precessing nonlinear spin waves are shown in Fig. 2. The black solid lines show the outline of the topological features of the nanocontact (ellipse) and the electrical connections. XMCD can clearly image the magnetic layer buried below (see Methods for details). The colour scale represents the size of the XMCD signal and corresponds to the out-of-plane precession angle (corresponding to the polar angle *θ* in spherical coordinates), proportional to the z-component of the magnetization. The magnetic contrast is observed only when a current *I*_DC_ is injected into the permalloy layer (providing the spin torque necessary to excite the spin wave) and when the frequency of the spin-wave excitation is locked by an alternating current *I*_mw_ synchronized to the X-ray pulses.

**Figure 3:**
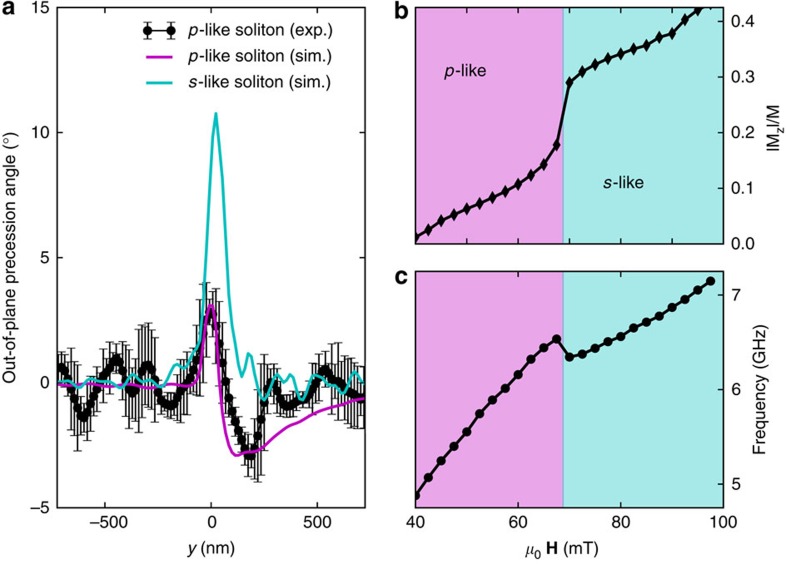
Analysis of the results. (**a**) Cross-section of experimental (scaled up a factor 7 in amplitude and averaged over three pixels along the *x*-direction), simulated *p*-wave and simulated *s*-wave solitons along the *y* direction and aligned with the nanocontact, as described in the text. The error bar at each point in the experimental cross-section represents the standard deviation of the signal in the three-pixel wide region. Such quantity represents the upper limit of the measurement error, as a similar fluctuation in the signal could be caused by magnetic moments effectively being misaligned from the average integrated signal. Simulated magnetic field dependence (**b**) of the out-of-plane component of the precessing magnetization (maximum value in proximity of the nanocontact) and (**c**) of the spin-wave frequency for an applied current *I*_DC_=8 mA. Pink (blue) areas indicate regions where *p*-wave (*s*-wave) solitons are excited.

The XMCD images in Fig. 2 demonstrate a time-dependent magnetic contrast evolving at time steps of 27 ps. (Note that the spin-wave frequency is 6.11 GHz, see Fig. 3. Also, a video rendering of the spin-wave dynamics is provided as Supplementary Movie 1). We observe that the magnetic contrast undergoes a sign change for a 180° phase shift, that is, three images apart, (a,d), (b,e), (c,f). Indications for this oscillation are also discernible further away from the nano-contact, although significantly closer to the noise floor of our experiment. Although panels (b), (c), (e) and (f) are in agreement with the expected *s*-like symmetry of localized nonlinear spin waves, the panels (a) and (d) display a departure from this picture. They show that the spin-wave develops a nodal structure with zero spin-wave amplitude located at *y*=100 nm from the centre of the nanocontact. Above and below this value, the magnetization points in opposite directions. This demonstrates that the excited spin wave, while being localized, is qualitatively different from the predicted spin waves with *s*-like symmetry. Instead, it resembles a localized excitation with *p*-like symmetry, which is only weakly localized around the nanocontact. We also find the centre of mass of this spin-wave motion to be displaced along the *y* axis by 100–150 nm with respect to the centre of the nanocontact, because of the magnetic potential well created by the superposition of applied, dipolar and Oersted fields.

### Micromagnetic simulations

We model the observed spin-wave motion with micromagnetic simulations using the fully three-dimensional, open-source MuMax code^24^, which can perform parallel calculations over the few thousand cores of a graphical processing unit. All simulation parameters are reported in the Methods section. Figure 2g–l shows the calculated spin-wave motion demonstrating excellent agreement with experiment. In particular, the simulations reproduce the nodal feature in the spin-wave amplitude at 0° and 180° phases. The spatial extent of the nonlinear spin waves describing the size of the transient potential well generated by the current-induced spin torque (see Fig. 3 and Discussion below for details) is another crucial feature that is well reproduced. We attribute the discrepancy in the out-of plane precession angle between experiment and theory to the spin-wave being phase-locked to the external microwave source only intermittently^25^,^26^ (see Methods for details). In addition, the simulations also reveal the presence of a propagating spin wave. This is identified as the second harmonics of the spin-wave emission, with a frequency of about 12 GHz, higher than the FMR frequency (around 7 GHz according to simulations) and hence with an allowed wavevector. This signal is not observed experimentally, probably because of the lower oscillation amplitude as well as the faster oscillation period (∼80 ps), comparable to the X-ray pulse duration (∼50 ps).

## Discussion

The agreement between experiment and simulations allows us to infer the key physical mechanims at play. The properties of the spin waves are qualitatively affected by the magnetic field landscape surrounding the nanocontact, caused by the vectorial superposition of applied, dipolar (from the patterned CoFe layer) and Oersted (from the current flowing through the nanocontact) magnetic fields. The effect of the combined magnetic fields is to create a potential well (that is, a field minimum) where the spin-wave can localize. Although simulations have predicted a similar localization mechanism^19^,^20^,^21^, this is the first time that a quantitative experimental observation is made, allowing us to determine the exact size and location of the excited spin wave.

Simulations also help understand the origin of the *p*-like symmetry of the excitation. Figure 2m–r shows the spatial map computed by micromagnetic simulations with a larger applied magnetic field *μ*_0_**H**=80 mT. The larger magnetic field causes the spin wave to strongly localize in the nanocontact region, with the expected *s*-like symmetry, and with larger precession amplitude. The qualitative difference between *s*- and *p*-like type spin waves is highlighted by computing the vertical cross-section of the simulated images of Fig. 2g, m across the nano-contact region, as shown in Fig. 3a. We performed detailed micromagnetic simulations as a function of applied field and we found that the transition between *s*- and *p*-like excitations is rather sharp, occurring in a field range *μ*_0_**ΔH**=2.5 mT, as shown in Fig. 3b,c. The *p*-like to *s*-like transition is evident in both the *z*-component of the magnetization (that is, in the out-of-plane precession angle), as well as in the spin-wave frequency. These simulations, performed at fixed bias current *I*_DC_=8 mA, suggest that the different extent of the spin-wave localization is due to the interplay between the torque caused on the ferromagnet by the spin transfer (that is, becaue of the current flowing through the nano-contact), and the torque induced by the total magnetic field acting on the magnetization. In turn, the extent of the localization region is the reason for the *s*- or *p*- like symmetry of the excitation, determined by the competition between exchange and dipolar interactions. A more localized excitation minimizes the exchange energy by preferring a *s*-like symmetry, whereas *p*-like states minimize the dipolar energy in more extended regions. More detailed considerations concerning the *p*-like to *s*-like transition (for instance its current dependence) are beyond the scope of this paper and will be the aim of a future work.

In conclusion, using the X-rays generated at a synchrotron lightsource, we have been able to record time-resolved images at the nanometre scale of the spin waves emitted by a nanocontact spin torque oscillator. These images allowed us to determine the detailed properties of the localized spin-wave excitation, a magnetic object with *p*-like character. Micromagnetic simulations closely reproduce the experimental evidence, and show that a *p*-like to *s*-like symmetry transition can be controlled by magnetic fields. Our study provides a deeper understanding of the nonlinear spin dynamics at the nanoscale, and offers a new degree of freedom for manipulating information in magnetic nanodevices.

## Methods

### Sample and experimental geometry

The sample considered here is a nanocontact spin torque oscillator. In this geometry, the excitation region is a 5-nm-thick Ni_80_Fe_20_ (permalloy) extended film, whereas the current injector is a patterned 150 × 50 nm^2^ Co_50_Fe_50_(8 nm)/Cu(8 nm) elliptical pillar with anisotropy axis 45° away from the applied field. These samples were fabricated with a process very similar to the one described in ref. 12. The only important difference is that our sample is grown on a 200-nm-thick SiN membrane substrate instead of a bulk Si wafer. SiN membranes transmit a large fraction of the incoming X-rays, allowing for the detection of the X-ray photons with a fast avalanche photodiode placed behind the sample. The schematic of the sample and of the measurement is shown in Fig. 1a.

### Experimental realization

The direct current applied to the sample was *I*_DC_=8.1 mA, and the injected microwave current *I*_mw_=0.8 sin(2*πft*) mA, that is, about 10% modulation of the direct current. This is, however, the upper boundary of the magnitude of the modulation, and we are likely closer to a frequency pulling regime rather than exact phase-locking^25^,^26^. An exact estimation of the magnitude of the injected microwave current is very challenging in our experimental conditions, where the microwave signal has to be transported through a vacuum chamber, propagate onto the sample carrier and finally reach the chip via wire-bonds. Multiple points of loss or impedance mismatch (hence of reflection) make precise calculations impractical. The frequency *f* of the microwave coincides with the spin-wave frequency *f*=6.11 GHz at the given direct current value (see Supplementary Fig. 1 for details). An external magnetic field *μ*_0_**H**=60 mT is applied along the horizontal axis of the images, as indicated by the arrow in Fig. 1a.

The time-resolved images of the spin-wave excitations were measured using a microwave synchronization board that we developed for this experiment. This board allows for the synchronization of a microwave signal generator with the Stanford Synchrotron Radiation Lightsource (SSRL) master clock at *f*_SSRL_=476 MHz. In turn, the signal from the microwave generator can be superimposed to the direct current that excites the spin waves, in order to realize injection locking between the phase of the microwave generator and the magnetization precession in the sample. This effect has been demonstrated by several groups in the past^26^,^27^.

The board realized a synchronization scheme similar to ref. 28. The microwave generator is synchronized to a frequency *f*_MW_=(*n*±1/*m*)·*f*_SSRL_ using a phase-locked loop electronics. Using a frequency offset at 1/*m*·*f*_*S*_ from the exact *n*·*f*_*S*_ harmonics will cause two subsequent photon bunches to probe two snapshots of the dynamical precession that are offset by 2*π*/*m* radians. At each *m*th event, the phase is offset by 2*π*, that is, it is back at the first phase offset. For the data presented here, *n*=13 and *m*=6, so that *f*_MW_=6.11 GHz. The different phases are stored in the different channels of a photon counter previously developed in our group^29^. Detection of the individual X-ray pulses (50 ps full-width at half-maximum (FWHM)) generated at SSRL was achieved using a biased avalanche photodiode (Hamamatsu S12426 Si-APDs) connected to two amplification stages. The synchronization jitter between the microwave signal and the storage ring (hence, not considering the intermittent locking intrinsic to the specific sample) is about 300 fs, measured as the voltage fluctuations of the signal created as the beating of the harmonic of the synchrotron clock with the signal from the microwave generator when the phase-locked loop is closed. Owing to the small synchronization jitter, the temporal resolution of the measurement technique is given only by the FWHM of the X-ray pulses. However, one can still resolve phases of the oscillation separated by a time step smaller than the X-ray FWHM as in our case, albeit with decreased contrast. This can be readily demonstrated computing the convolution between a sinusoidal and a Gaussian curves, which is the quantity that one actually measures in the experiment.

Finally, we also implemented a second modulation scheme to synchronize the excitation signal with the orbit clock of the storage ring *f*_orbit_=1.28 MHz. This allowed us to use the odd and the even orbits of the synchrotron to alternatively record the signal and, respectively, the reference data with minimum delay, greatly suppressing the effect of drift in the synchrotron intensity in our measurements. Other experimental parameters of the experiment were as following: photon flux through the sample of the order of 10^9^ ph s^−1^, spectral bandpass *E*/Δ*E*∼5,000 and nominal spot size ≈35 nm, computed from the Rayleigh criterion 1.22Δ*r* for a zone-plate with outer zone-width Δ*r*=30 nm. Acquiring six images such as the ones presented in Fig. 2a–f, consisting of 2,500 pixels (50 × 50, with a step size of 30 nm) each, takes ∼8 h. Further details of our measurement technique can be found in an upcoming publication.

### Micromagnetic simulations

Numerical simulations were performed using a MuMax code^24^. We considered a two-dimensional layer and integrated the Landau–Lifshitz–Gilbert–Slonczewski equation to describe the magnetization dynamics. A current density is taken in the area of the ellipsoidal nanocontact region (with the same nominal dimensions of the nanocontact) and we computed in a much larger area of ∼2 μm^2^ with 4 nm resolution. We implemented absorbing boundary conditions to avoid the effect of spin-wave reflection from the edges. We tested different absorbing conditions and different sizes of the simulated systems (2 × 2, 4 × 4 and 8 × 8 μm^2^) and found no impact on the presented results.

We considered an effective field that includes contributions from demagnetizing, exchange, Zeeman and Oersted fields. For the calculation of the Oersted field, we considered an infinite ellipsoidal rod (in the direction parallel to the flow of current) with the dimensions of the nanocontact, and we solved the integral 
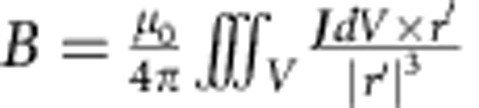
 in two steps (**J** is the current density vector and *r*′ is the displacement vector from the centre of the wire): at first, we integrated over one of the ellipse dimensions using the software Wolfram Mathematica, obtaining an analytical expression; then, we performed a numerical integration of that analytical expression (using MuMax) along the other dimension of the ellipse. We also note that we included the stray fields from the patterned ellipse of Cobalt Iron (CoFe) from the polarizing layer. Thermal effects and crystalline anisotropy are neglected. For the free magnetic layer, the following parameters were used: saturation magnetization *M*_*s*,P*y*_=670 × 10^3^ A m^−1^, Gilbert damping constant *α*=0.01, and exchange constant *A*=1.3 × 10^11^ J m^−1^. (We have found that neither the exchange constant nor the damping parameter affect the qualitative simulation results in a significant way.) The value of the saturation magnetization *M*_s,P*y*_ is 15–20% smaller than the nominal value and allows our simulations to reproduce the experimental excitation frequency. Such smaller value could be caused by interdiffussion of Cu atoms into the thin Py layer or by local heating in the nanocontact area, which is supposed to be greater than 100 °C (ref. 30), although there is not a clear consensus in the community on this issue. We notice that even smaller values have been measured by vibrating sample magnetometer and used in micromagnetic studies in nanocontacts very similar to ours, where the torque was provided through a Cu/Py interface^31^. The saturation magnetization *M*_s,CoFe_ for the CoFe polarizing layer was set to 1,530 × 10^3^ A m^−1^. An oscillating current is also injected to the dc current that allows frequency lock-in: we used a modulation of the dc current of 15%. We include the full simulation code for the images presented in Fig. 2 as Supplementary Note 1. The simulated spin-wave frequencies are typically within 1% of the experimental value.

## Additional information

**How to cite this article:** Bonetti, S. *et al.* Direct observation and imaging of a spin-wave soliton with *p*-like symmetry. *Nat. Commun.* 6:8889 doi: 10.1038/ncomms9889 (2015).

## Supplementary Material

Supplementary Information Supplementary Figure 1 and Supplementary Note 1

Supplementary Movie 1 Movie rendering of the experimental spin-wave dynamics presented in Fig. 2(a)-(f)
