# Supplementary material for: Direct observation and imaging of a spin-wave soliton with p-like symmetry
Source: Nat Commun. 2015 Nov 16;6:8889. doi: 10.1038/ncomms9889 (PMC4660209; doi:10.1038/ncomms9889)
Supplement: Supplementary Information — Supplementary Figure 1 and Supplementary Note 1 [file ncomms9889-s1.pdf]

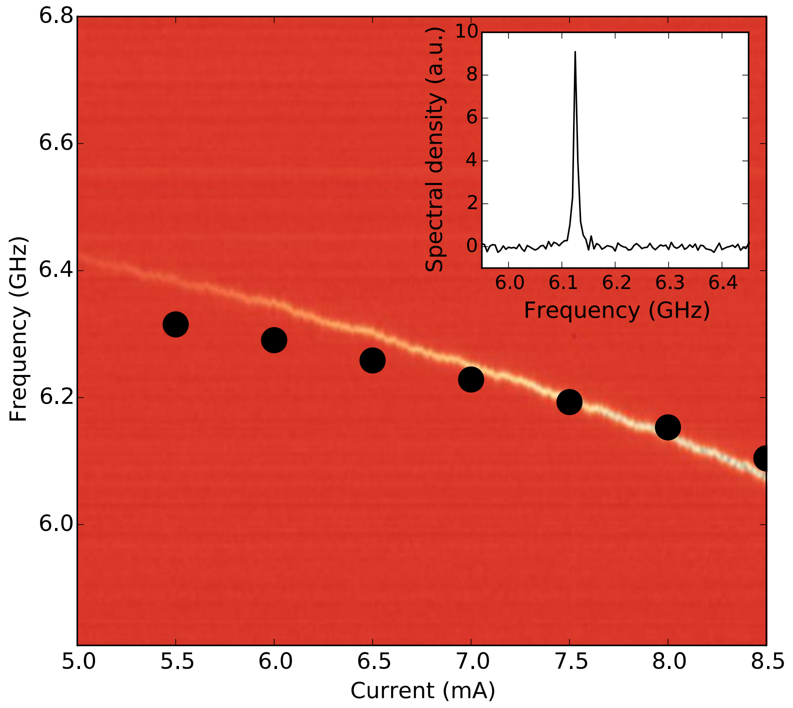

Supplementary Figure 1 Power spectral density map of the frequency vs current characteristics of the spin waves emitted by the nanocontact spin torque oscillator, measured with a spectrum analyzer while the sample was mounted in the microscope vacuum chamber under a static magnetic field $\mu_0 H$ = 60 mT. Symbols: oscillation frequencies retrieved from micromagnetic simulations. The most likely cause of discrepancy between calculated and experimental values is the modeling of the current flow in the nanocontact. Our simulations are considering an ideal flow of current (both electrically for the Oe fields and magnetically for the spin transfer torque), and does not account for the diffusive electric transport at the interface. Inset: power spectral density of the emitted spin wave for I = 8.1 mA, showing the narrow linewidth ≈ 10 MHz) of the emitted spin waves, confirming that the fabrication of the sample on free-standing SiN membranes did not affect the quality of the oscillator.

Supplementary Note 1

Supplementary Material: mumax3 code
Authors: Ferran Macìa and Joan Manel Hernàndez

All the micromagnetics were run with the default method in mumax that is a Runge-Kutta 45 (Dormand-Prince) solver with variable step. All the information on this is available online in the mumax webpage (http://mumax.github.io/api.html). In the following code we indicate a run time of 40 ns, which corresponds to computations lasting for around 10 minutes in a computer with a 3000-core gpu. This is a reasonable time to start setting up these simulations, but longer run times may be needed depending on initial conditions and close to transitions. In the computations presented in this work we always used much longer run times, although in most of the cases we could observe a stable solution already after a few tens of nanoseconds.

```
// mumax3 is a GPU-accelerated micromagnetic simulation open-source software
// developed at the DyNaMat group of Prof. Van Waeyenberge at Ghent University.
// The mumax3 code is written and maintained by Arne Vansteenkiste.


//Grid definiton
LL:=512
SetGridSize(LL, LL, 1)
SetCellSize(4e-9, 4e-9, 5e-9)
SETPBC (4,4,0) // Boundary conditions

deg:=pi/180.

// External parameters
h_value:=0.060 // External field value (Tesla)
h_angle:=45 * deg // External field orientation
pola:=0.45 // Current polarization
pol_angle:=10*deg // Direction of the polarization of the current.
// It comes from the orientation of the polarizing
// layer (an ellipse) under a 60 mT field at 45 deg
curr:=-0.01 // Current passing through the contact (A)
freq:=6.165e9 // Modulation frequency
modulation:=0.15 // This is the amplitude of the modulation
// with respect to the applied current

// Material parameters. Default values and the ones that will apply to the free layer.
Msat = 670e3 // Saturation magnetization of free layer
Aex = 13e-12 // Exchange stiffness constant of free layer
alpha = 0.01 // Damping constant
lambda = 1 // Asymmetry
epsilonprime = 0 //
temp = 0. // temperature (non zero temperatures
// require different PDE solver)

//Ellipse geometry
R_a := 75e-9 // (m)
R_b := 20e-9 // (m)
A_circ := pi*R_a*R_b // (m^2)

setGeom(universe())

//Definition of contact geometry (region 1)
DefRegion(1, ellipse(2*r_a,2*r_b))

//Absorbing boundary conditions (regions 2, 3 and 4)
DefRegion(2,square(LL*4e-9 * .85).inverse())
DefRegion(3,square(LL*4e-9 * .9).inverse())
DefRegion(4,square(LL*4e-9 * .95).inverse())
alpha.setregion(2,.05) // large damping constant in region 2
alpha.setregion(3,.15) // larger damping constant in region 3
alpha.setregion(4,.45) // even larger damping constant in region 4


//Setting the initial state (Magnetization along the applied field)
m = Uniform( cos(h_angle) , sin(h_angle) , 0.)

// Setting the polarization in the nanocontact (region 1)
px := cos(pol_angle)
py := sin(pol_angle)
```

```
pz:= 0
fixedlayer.SetRegion(1, vector(px, py,pz))

// save the magnetization below the contact
tableadd(m.region(1))

// DC external magnetic field
B_ext = vector( h_value *cos(h_angle), h_value *sin(h_angle), 0.)

// Dipolar field created by the fixed layer
// (precalculated externally by another code).
// The fixed layer is not simulated in the present code.
B_ext.add(loadfile("dip_field_L5.ovf"), 1.)

//Let the system relax from the initial state to get the equilibrium
// before exciting it with the current.
relax()

// Let us switch on the current.
// The current produces the spin transfer torque
pol.setregion(1,pola)
curr=-0.0081
j.SetRegion(1,vector(0,0, curr/A_circ *(1.+modulation*sin(2 * pi* freq*t))))

// The current creates an Oersted field distribution around the contact.
// We load the precomputed Oe fields for a 1 Amp/m^2 current density.
// It should be scaled to the correct current value
B_ext.add(loadfile("Oe_f_1L.ovf"), curr/A_circ*(1.+modulation*sin(2 * pi* freq*t)))

tableautosave(2e-12)
run(4e-8)
```
